# Melatonin supplementation counteracts fiber loss in knee ligaments of diabetes-induced rats

**DOI:** 10.3389/fphar.2024.1399719

**Published:** 2024-07-29

**Authors:** Olga Adamska, Artur Wnuk, Agnieszka Kamińska, Małgorzata Poniatowska, Bartosz Maciąg, Maciej Kamiński, Artur Stolarczyk, Maima Matin, Atanas G. Atanasov, Marcin Łapiński, Artur Jóźwik

**Affiliations:** ^1^ Department of Ophthalmology, Collegium Medicum, Cardinal Stefan Wyszynski University, Warsaw, Poland; ^2^ Hospital in Ostrow Mazowiecka, Ostrów Mazowiecka, Poland; ^3^ Department of Nuclear Medicine, Pomeranian Medical University in Szczecin, Szczecin, Poland; ^4^ Orthopedic and Rehabilitation Department, Medical University of Warsaw, Warsaw, Poland; ^5^ Medical Faculty, Lazarski University, Warsaw, Poland; ^6^ Institute of Genetics and Animal Biotechnology of the Polish Academy of Sciences, Magdalenka, Poland; ^7^ Laboratory of Natural Products and Medicinal Chemistry (LNPMC), Center for Global Health Research, Saveetha Medical College and Hospital, Saveetha Institute of Medical and Technical Sciences (SIMATS), Chennai, India; ^8^ Ludwig Boltzmann Institute Digital Health and Patient Safety, Medical University of Vienna, Vienna, Austria

**Keywords:** ligaments alteration in diabetes mellitus, diabetes mellitus, ligaments alteration, orthopedic complications, diabetes ligaments

## Abstract

Diabetes mellitus (DM) is a prevalent metabolic disease. The clinical impact of sustained hyperglycemia on ligament healing has not been well characterized. Diabetes is a known cause of macro-, microvascular, and diabetic ulcer healing difficulties among tissues. Therefore, we aimed to investigate the healing potential occurring in injured and healthy ligaments among diabetic and healthy individuals using a rat model. We hypothesize that DM may contribute to altering the knee medial collateral ligament (MCL), thus its morphology, biochemical fitness, and functionality. The study cohort consisted of 40 rats. The animals were randomized into four equal groups. Groups I and II (20 rats) received saline subcutaneously and served as controls. Groups III and IV (20 rats) were injected with a single dose of streptozotocin (STZ). All animals underwent surgery to cut the left tibial collateral ligament in the hind limb and suture it. The access site was sutured to create inflammation and study the regenerative capacities of animals with normal carbohydrate metabolism and pharmacologically induced diabetes. Each animal then underwent sham surgery to access and suture the right tibial collateral ligament in the hind limb without ligament intervention. After the animals had undergone surgeries, groups II and IV were given melatonin supplementation for 4 weeks. Rats with DM presented with more fibrosis and calcification of the MCL and decreased healing potential. Treatment with melatonin in diabetic rats mitigated alterations and improved the antioxidant status of ligaments from the diabetic group.

## 1 Introduction

Ligaments play a unique and important role in the musculoskeletal system. However, the knowledge about the molecular mechanisms in the connective tissue is not as advanced as knowledge about other tissues (Longo et al., 2009; Leong et al., 2020). This is partly because, until recently, the transcription factors crucial for the formation and maintenance of ligaments remained unknown, and there is still much to discover (Nelson et al., 2006). Understanding the pathogenesis of non-insulin-dependent diabetes mellitus and the development of more effective therapies may be facilitated by the establishment of animal models with pathophysiology closely resembling human disease (Fleming et al., 2005). Tendons and ligaments arise from different developmental processes. The regulatory pathways and morphologies of the two tissues predispose them to play heterogeneous roles (Fleming, 2003). Therefore, the available findings in tendinopathies are not well adopted in the concept of ligament regeneration and require further study.

Systemic diseases with long-lasting inflammation impact the organism in various ways (Fleming, 2003; Stolarczyk et al., 2018). Diabetes mellitus (DM) is a prevalent metabolic disorder that impairs barrier functioning and healing responses throughout the human body. In particular, DM inhibits the expression of mitogenic growth factors and simultaneously stimulates that of pro-inflammatory cytokines through epigenetic mechanisms. Moreover, hyperglycemia and oxidative stress induced by DM prevent the expansion of mesenteric cells that are involved in both soft and hard tissue wounds (Hill et al., 2005; Akbarzadeh et al., 2007; Mullaji et al., 2008). Those mechanisms can be investigated in animal models, and further comprehensive studies are needed to address the issues. Therefore, research on ligament alteration is of great significance.

Not many researchers correlate the damage to ligaments with possible systemic diseases and instead focus on trauma as the cause of damage (Akbarzadeh et al., 2007; Mullaji et al., 2008; Parasuraman et al., 2010; Bicer et al., 2011). However, DM is one of the most common and debilitating medical conditions (Fleming, 2003; Stolarczyk et al., 2018).

Patients with DM may experience increased surgical risks, including a higher risk of infection, compromised tissue quality for repair, and a propensity for problems with wound healing (Sotoudeh and Namavar, 2022).

A major interest of this study was to compare both the regenerative abilities of ligament and skin injury in diabetic rats and the regenerative abilities of ligament and skin injury among nondiabetic rats, as well as the putative benefits of melatonin supplementation. We hypothesize that DM may contribute to the impaired healing potential of the knee medial collateral ligament regardless of the extent of the damage. The sacrifice of rats in the present study enhanced the current state of the art by collecting tissues for microscopic evaluation. Furthermore, the study aimed to identify alterations in the microarchitecture of MCL and remodeling of the molecules for insights into the treatment of patients presenting with DM and musculoskeletal complications. Ligament recovery is demanding because of its multifactorial role in the musculoskeletal system. The healing of the joint soft tissues in comorbidity with diabetes is challenging in clinical practice. Accumulated evidence and data from the present study support future preclinical and clinical experiments. Melatonin’s beneficial effects on insulin signaling and lipid metabolism in obesity, as well as decreasing inflammation and antioxidant activity in diabetes and related comorbidities, have been proved. This is important due to the abundance of patients with obesity in clinical practice. DM and obesity are common comorbidities in the orthopedic department, and the result again stresses the need for holistic therapy for soft tissue damage in patients and the role of sleep and melatonin at high levels. The results provide insights into the pathogenesis of ligament alteration in diabetes and uncover melatonin’s potential for downregulating inflammation, oxidative stress, and inhibitors of systemic metabolism.

## 2 Materials and methods

Male Sprague–Dawley rats with a weight ranging from 280 to 300 g, 12 weeks of age, were purchased from the Central Laboratory of Experimental Animal Models, Medical University of Warsaw, Warsaw, Poland. The rats were kept in plastic cages with metal lids, with two rats per cage. The animals received a commercial diet and had free access to water. The rats were placed in a conventional laboratory housing with a 12 h light/12 h dark cycle at a temperature of 21°C ± 2°C and 55% ± 10% humidity.

All the animals received housing conditions according to the criteria outlined in the Guide for the Care and the Use of Laboratory Animals prepared by EU Directive 2010/63/EU for animal experiments. The ethical regulations were followed in accordance with national and institutional guidelines for the protection of animal welfare during experiments. The study was designed according to the ARRIVE guidelines.

The animals used in the study were randomly separated into four groups.

Group I (n = 10) general control group: the group was not induced with DM and fed on a normal diet.

Group II (n = 10) melatonin-supplemented control group: the group was fed a normal diet and supplemented with 3 mg/kg/day melatonin *per os* (p.o.) for 4 weeks (Bicer et al., 2011).

Groups III and IV were injected with streptozotocin (STZ) at a dose of 60 mg/kg of body weight, dissolved in a freshly prepared buffer (0.1 mol/L citrate, pH 4.5). The rats were fasted for 8 h before the STZ injection. The solution was injected into rats intraperitoneally (i.p.), with the aim to induce diabetes (Akbarzadeh et al., 2007). Fasting blood glucose level was assessed on the day of the intraperitoneal injection of STZ and 72 h after the injection, and a venous blood glucose test was performed by glucometer strips. The animals were immobilized in a plastic tube and placed on the heating platform to dilate the vessels in their tails. A drop of blood was obtained by puncturing the vein with a needle (Parasuraman et al., 2010).

Rats with consistent blood glucose levels ≥200 mg/dL 72 h after the STZ injection were regarded as successful diabetic models. To reduce the risk of mortality caused by STZ, the animals used in the study were relatively young. No animal died, and no animal experienced an ineffective DM induction.

Group III (n = 10) diabetic group: the group in which DM was induced by an i.p. injection of 60 mg/kg STZ and was not supplemented with melatonin.

Group IV (n = 10) melatonin-supplemented diabetic group: the group in which DM was induced by an i.p. injection of 60 mg/kg STZ and was supplemented with 3 mg/kg/day melatonin p. o. for 4 weeks (Bicer et al., 2011).

### 2.1 Tissue preparation

**FIGURE 1:**
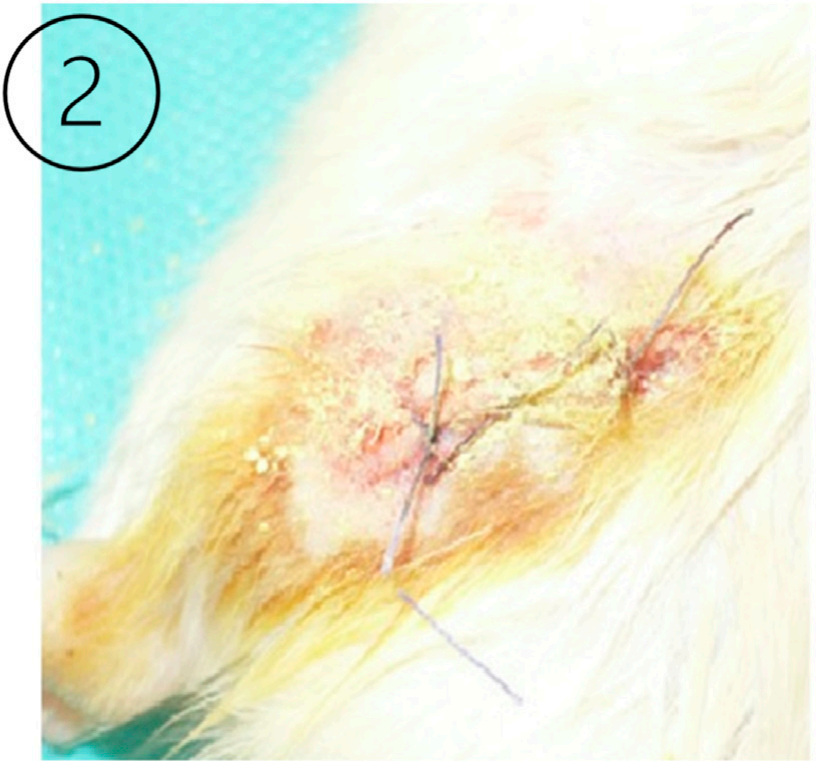
The right image (Leong et al., 2020) shows the animal model knee joint surgical site during the ligament preparation procedure.

**FIGURE 2:**
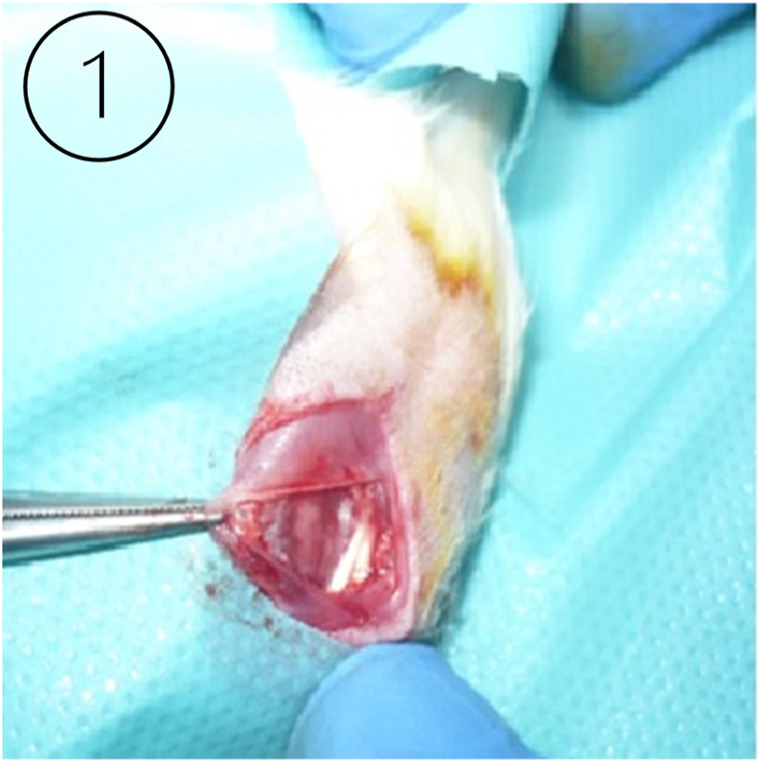
The left image (Longo et al., 2009) shows a sutured surgical wound in the experimental animal model after the intervention in the knee joint ligament.

A surgical dissection of the MCL was performed on the left lower extremity of an animal, and a sham procedure was performed on the right lower extremity of the same animal to simulate the surgical conditions in surrounding tissues and to compare the process of regeneration. The procedure was performed under general anesthesia (ketamine 75 mg/kg of body weight + xylazine 7 mg/kg of body weight) (Sotoudeh and Namavar, 2022). After disinfection, a longitudinal cut from 3 mm below the knee joint to 3 mm above the joint was made, then the skin and fascia were cut to reveal the MCL (Sairyo et al., 2007) (Figure 1). The ligament was dissected and cut in the middle. Fascia, ligaments, and skin were closed with a surgical suture (Vicryl 6.0, Ethicon) (Figure 2). A sham operation was performed on the right limb. All steps were the same as the operation of dissecting the left MCL, except that the ligament remained intact after preparation. Post-operative care and pain relief (paracetamol in drinking water 300 mg/kg of body weight) were provided for 2 days from the day of surgery and possibly repeated in the case of pain distress caused by the operation. Animals received 2.5% Baytril antibiotic prophylaxis (enrofloxacinum) s.c. mg/kg of body weight dose for a period of 3 days post-operatively (Trouchon and Lefebvre, 2016; Sotoudeh and Namavar, 2022).

**FIGURE 3:**
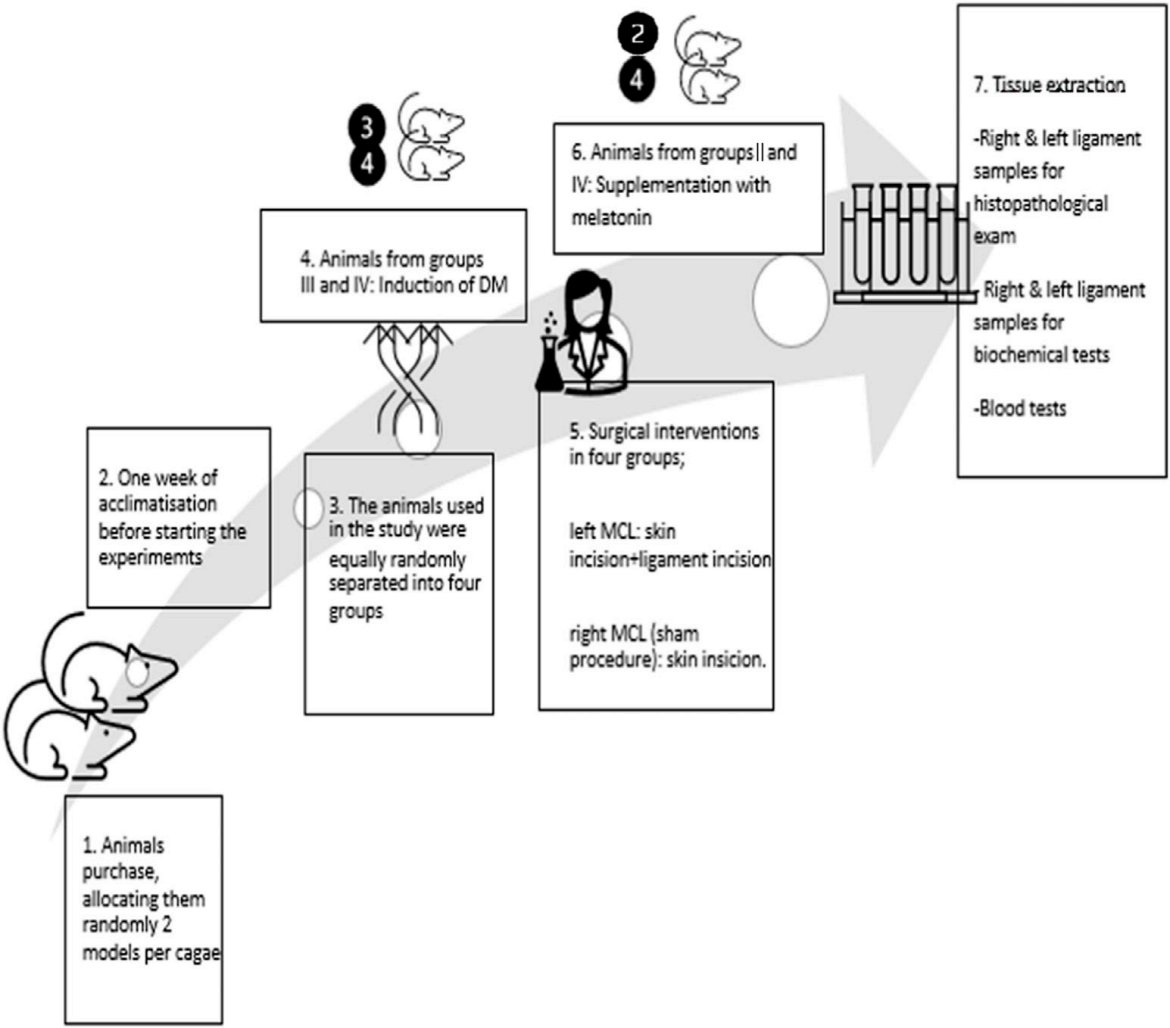
Schematic chronologic representation of the study design.

Table 1 provides a summarized division of the animals into the four groups, each with 10 models. Figure 3 represents the schematic overview of the study design.

**TABLE 1:** Experimental groups.

Group	Surgical intervention	Additional interventions	Number of rats
Group I	Normoglycemia/one-sided surgical dissection of the left MCL/simulated surgical dissection of the right MCL (sham procedure) Total resection of the MCL that was surgically dissected (28 days post-operatively)	—	10
Group II	Normoglycemia/one-sided surgical dissection of the left MCL/simulated surgical dissection of the right MCL (sham procedure) Total resection of the MCL that was surgically dissected (28 days post-operatively)	Oral melatonin supplementation for 4 weeks starting after the surgical procedures	10
Group III	DM/one-sided surgical dissection of the left MCL/simulated surgical dissection of the right MCL (sham procedure) Total resection of the MCL that was surgically dissected (28 days post-operatively)	—	10
Group IV	DM/one-sided surgical dissection of the left MCL/simulated surgical dissection of the right MCL (sham procedure) Total resection of the MCL that was surgically dissected (28 days post-operatively)	Oral melatonin supplementation for 4 weeks starting after the surgical procedures	10
	Summary		40

### 2.2 Controlled treatment with melatonin supplementation

Melatonin (Mel) (Sigma M-5250) supplementation was given to the animals in groups II and IV at a dose of 3 mg/kg/day for 4 weeks after the surgical procedures (Bicer et al., 2011).

### 2.3 Tissue acquisition and preparation

The operating surgeon obtained tissue from the central portion of the MCL (entire layer) to minimize tissue damage and optimize the harvest of the ligament only. After all fat tissue was removed, the tissue specimens were washed with saline to remove blood and bodily fluid contaminants. All specimens were transported under sterile conditions to the laboratory within 20 min of excision. The harvesting and processing procedures were identical in all rats. Two experienced scientists and the first author performed the surgical interventions.

The specimens were fixed in 4% formaldehyde in phosphate-buffered saline and prepared for paraffin embedding. Twenty consecutive sections, each 4-µm thick, were cut on a microtome and treated with the appropriate stains. A light microscope (Nikon, Japan) was used to view and photograph the specimens.

### 2.4 Fibrosis and elastin fiber loss grading

Four cross sections of each specimen were randomly selected for histologic analysis and graded independently by four of the authors (A.S., A.W., M.P., and O.A.), all of whom were blinded to the group. The severity of fibrosis (based on Masson trichrome staining) was graded on four-point scale, according to the guidelines set by Sairyo et al. (2007), Ding et al. (1988): 0—normal tissue showing no fibrotic region; grade 1—fibrosis of less than 25% of the entire area; 2—fibrosis of 25%–50% of the entire area; grade 3—fibrosis of 50%–75% of the entire area; 4—more than 75% fibrosis. The same method was used to evaluate elastin fiber loss (based on elastica van Gieson and Orcein staining). Average grades of fibrosis and elastin fiber loss were calculated for every sample. The results are presented in Figure 3.

### 2.5 Tissue homogenates

Rats from all groups were terminally anesthetized after 6 weeks by an intraperitoneal injection of 1.5–2 mL/kg (199.95–267 mg/kg) of body weight of sodium pentobarbital + 40.05–53.4 mg/kg of body weight of pentobarbital. Blood samples were drawn by cardiac puncture into tubes containing EDTA. Ligament samples were also collected from the different experimental groups for histopathological and biochemical examination.

In the first part, ligament harvesting was done immediately, and the collected tissue was homogenized in a volume/tissue ratio of 100 mM phosphate buffer (pH 7.4) containing 22 mg% EDTA. Organs from different experimental groups were fixed in 10% buffered formalin for 24 h. After fixation, tissues were washed in tap water, then dehydrated in an ascending series of 70%, 90%, 95%, and 100% ethanol, followed by clearing in xylene and embedding in paraffin wax at 55°C. Five sections (4 µm thickness) were cut from each tissue, followed by staining with hematoxylin and eosin. In brief, slides were put in xylene to dissolve the paraffin wax, then in absolute alcohol for 2 min, 95% alcohol for 1 min, and 70% alcohol for 1 min. Slides were stained with hematoxylin for 5 min, then washed in tap water. Excess stain was removed in acid alcohol for a few seconds. Sections were stained with eosin for 4 min to 5 min, washed in water, and then washed in absolute alcohol (95%) and xylene.

The tissue homogenate was then stored at −20°C for determination of lipid peroxides, total thiols, activity of catalase, superoxide dismutase (SOD), and glutathione S-transferase (GST). The ligament homogenates were retrieved to analyze the concentrations of oxidative biomarkers: tissue total antioxidant status (TAS), total oxidant status (TOS), ROS activities, and oxidative stress index (OSI). Lipid peroxidation was determined using malondialdehyde (MDA) and glutathione (GSH). Blood samples were centrifuged to separate plasma and tested for levels of fasting blood glucose, total lipid, triglyceride levels, cholesterol levels, lipid peroxides, nitric oxide (NO), total thiols, and total albumin and activities of catalase, uric acid, SOD and GST.

### 2.6 Biochemical analyses

#### 2.6.1 Lipid peroxidase and nitric oxide

Lipid peroxide concentration was detected in obtained plasma and tissue homogenates. The product of the reaction between malondialdehyde and barbituric acid was measured. NO was determined in plasma as nitrite concentration after the reduction of nitrate to nitrite. The reaction took place at 22°C for 20 min, and the absorbance at 546 nm was measured using an NaNO_3_ solution as the standard (Ding et al., 1988).

#### 2.6.2 SOD, catalase, GST, MDA, and ceruloplasmin activities

SOD activity was measured in three tubes: plasma, hemolysates, and tissue homogenates, and was calculated using its inhibitory activity of the autooxidation of epinephrine in an alkaline medium (Misra and Fridovich, 1972). GST activity in hemolysates and tissue homogenates was measured through chemical determination using 1-chloro2,4-dinitrobenzene as the substrate (Habig et al., 1974). Catalase activity in hemolysate and tissue homogenates was determined based on its ability to decompose H_2_O_2_ and measured at 240 nm (Luck and Bergmeyer, 1963). Serum MDA was determined by a rat MDA ELISA Kit (MBS268427, MyBioSource, United States). SOD activity was determined by a rat SOD ELISA Kit (MBS266897, MyBioSource, United States). A Bio-Rad protein assay was used to measure total protein concentration in tissue homogenates. Ceruloplasmin activity was determined using a para-phenylenediamine dihydrochloride method (Schosinsky et al., 1974).

#### 2.6.3 Total plasma thiols, uric acid, and albumin

Total thiols were detected using the chemical method described by Ellman (1959). Albumin detection was performed colorimetrically with a commercial kit (Sclavo Diagnostics, Italy). Uric acid was determined by enzymatic colorimetric method using a commercial kit (Biocon, Burbachy, Germany).

#### 2.6.4 Plasma cholesterol, triglycerides, and total lipids

Cholesterol concentration was determined using an enzymatic colorimetric kit (Biocon). Triglyceride concentration was measured using an enzymatic hydrolysis of triglycerides with subsequent determination of liberated glycerol by colorimetry (Boehringer). Total lipids were chemically determined by the phosphovanillin method (Knight et al., 1972).

#### 2.6.5 Total protein and blood glucose

Total proteins in plasma and tissue homogenates of ligaments and muscles were determined by a biuret tartrate method using a commercial kit (Sclavo Diagnostics). Blood glucose level was determined by a commercial kit (Biocon).

#### 2.6.6 Oxidative status

Ligament harvesting was done, and bone samples were excised and homogenized in cold 0.9% NaCl using glass equipment to obtain a 10% (w/v) homogenate. The homogenates were centrifuged at 10,000×*g* for 15 min at 4°C so as to obtain the necessary clear supernatants required for the experiments. TAS, TOS, and ROS activities and OSI were assessed using the method described by Erel (2004) and Erel (2005).

#### 2.6.7 Lipid peroxidation parameters

MDA analyses were performed in the bone tissue as mentioned earlier, and the levels were given as nmol/g/protein (Draper and Hadley, 1990). The glutathione GSH levels in the bone tissue were determined, as mentioned earlier. The GSH level was determined as mg/dL/g protein (Atroshi et al., 1981).

### 2.7 Statistical analysis

The collected data were analyzed using the STATISTICA 13.3 TIBCO Software, Inc., statistical package. Data are expressed as mean ± SD for all parameters in plasma, erythrocyte lysate, and tissue homogenates. The evaluation of the distribution of the analyzed variables was verified with the Shapiro–Wilk test. When two dependent variables were compared, and the distribution was consistent with a normal distribution, Student’s t-test was used. When comparing the two dependent variables and non-compliance of the distribution with the normal distribution, the Wilcoxon pair order test was applied.

In the case of multiple independent samples (such as a comparison of animal models from different groups) with a distribution that did not match the normal distribution, the Kruskal–Wallis ANOVA test was used. *Post hoc* group comparisons were carried out using the Newman–Keuls test. To determine the occurrence of statistically significant differences between specific variables, multiple comparisons of average ranks for all trials (such as a comparison of parameters from a single-animal model) were used. Trait correlations were assessed using the Spearman test:

• Rxy > 0 positive correlation—when the value of X increases, so does Y,
• Rxy = 0 no correlation—when X increases, Y sometimes increases and sometimes decreases,
• Rxy < 0 negative correlation—when X increases, Y decreases.

The correlation strength was determined according to the following guidelines:

• Rxy = 0 variables are not correlated
• 0 < Rxy < slight correlation
• 0.1 < Rxy < 0.3 weak correlation
• 0.3 < Rxy < 0.5 average correlation
• 0.5 < Rxy < 0.7 high correlation
• 0.7 < Rxy < 0.9 very high correlation
• 0.9 < Rxy < 1 almost complete correlation

The statistical significance level of *p* < 0.05 was adopted.

## 3 Results

### 3.1 Histological screening of stained samples: Sairyo score assessment of the fibrosis of knee ligament samples

The values presented in the tables are compared in the following manner: group I is a control group for groups II and III, and group II is a control group for group IV.

The left MCLs that underwent the surgical procedure presented with worsened overall status compared to the right MCLs that experienced a sham procedure (Table 2). Specifically noticeable was significant fibrosis in the left MCL compared to the right MCL in group III of animals with a DM score of 3.43 ± 0.88; *p* < 0.05 and in group I animals with a DM score of 1.44 ± 0.7; *p* < 0.05. The third section (a Sairyo score of 1.44) of dissected ligament tissue from the normoglycemic group appeared fibrotic, and 75% of the MCLs dissected from diabetic rats were fibrotic. Increased loss of elastin fiber was observed in the dissected MCLs from all groups, *p* < 0.05. Group II, the melatonin-supplemented control group, had less elastin fiber loss than group I. Right MCLs from group II achieved 0 in Sairyo Score and right MCLs from the group I achieved 0.5 ± 0.2, *p* < 0.05. Left MCLs from group II achieved 0 in Sairyo Score and left MCLs from the group I achieved 1.05 ± 0.55, *p* < 0.05. The right MCL samples from all groups showed statistically significant more calcification than the tissue retrieved from the left MCL from the same groups. The nondiabetic group presented with calcification in the dissected left MCL (1.05, ± 1.7) and no sign of calcification among the right MCL with statistical significance (*p* < 0.05).

**TABLE 2:** Analysis of animals by group.

	Group I: Control/Right MCL n = 10	Group I: Control/Left MCL n = 10	Group II: Control+M Right MCL n = 10	Group II: Control+M Left MCL n = 10	Group III: DM/Right MCL n = 10	Group III: DM/Left MCL n = 10	Group IV: DM+M/Right MCL n = 10	Group IV: DM+M/Left MCL n = 10
Sairyo score	0	1.44 (±0.7)	0.25 (±0.3)^a^	0.5 (±0.11)^a^	2.25 (±0.5)^a^	3.43 (±0.88)^a^	0.5 (±0.15)^b^	0.75 (±0.05)^b^
Elastin fiber loss	0.5 (±0.2)	1.05 (±0.55)	0^a^	0^a^	0.7 (±0.2)^a^	2.3 (±0.9)^a^	1.25 (±0.2)^b^	1.0 (±0.1)^b^
Calcifi-cation	0	1.05 (±1.7)	0^a^	0.25 (±0.05)^a^	0.67 (±0.2)^a^	1.97 (±0.28)^a^	1.0 (±0.35)^b^	0.96 (±0.14)^b^

Abbreviations: Control, control group; Control + M, control group + melatonin supplementation; DM, diabetic group; DM + M, diabetic group + melatonin supplementation; Right MCL, sham procedure was performed; Left MCL, incision of the ligament was performed. Data are expressed as mean ± SD. ^a^ *p* < 0.05 versus the control group; ^b^ *p* < 0.05 versus the diabetic group.

**FIGURE 4:**
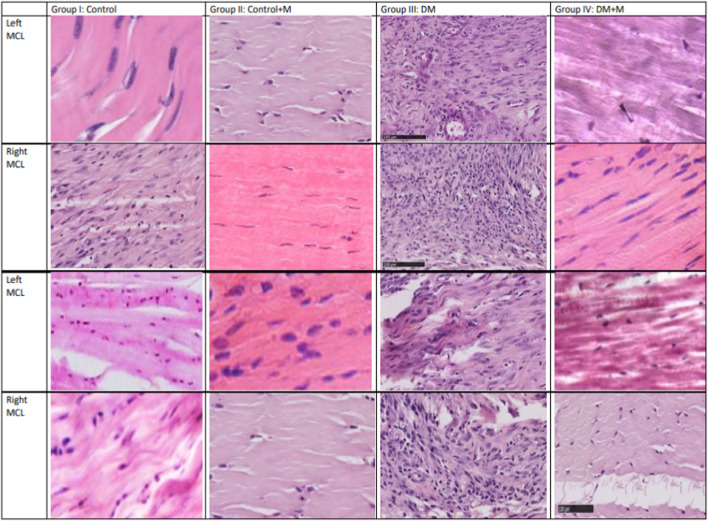
Histology-stained sections (longitudinal dissection) from the four groups: normoglycemia and diabetes mellitus, with and without melatonin supplementation. Each column contains light microscopy images obtained from a single representative model and from the same section. A scale bar is shown on the left bottom corner (100 µm). Each row is indicated for one model from which the tissue was retrieved. Sample imaging was used to assess the status of the tissue. The right MCL comes from the extremity on which the sham procedure was performed, and the left MCL comes from the extremity on which a ligament incision was performed. Quantitative results are presented in Tables 3, 4.

Specifically, the specimens belonging to group III, animals with DM, present with hypercellularity and fibrosis compared to the integrated, linear fibroblasts in the control group. What is interesting is that group IV, animals with DM receiving supplemental melatonin, presented with an improved overall quality of the dissected ligaments (Figure 4).

### 3.2 Microscopic histopathological assessment: optic quantitative screening for alterations in the extracted ligament samples from different experimental groups

All (100%) of the connective tissue of the right and left MCL samples from group I, normoglycemic animals, appeared with typical ligament components with statistical significance (*p* < 0.05) (Table 3). An assessment of the remaining groups indicated that in group II of animals without DM receiving Mel supplementation, the right MCL appeared with a characteristic pattern in each sample with statistical significance (*p* < 0.05). In the left MCL, most samples (8; *p* < 0.05) appeared to be typical, but two samples presented with single lymphocytic infiltration, loosened structure, mast cell infiltration, and increased fibroblasts compared to group I (*p* < 0.05).

**TABLE 3:** Optical quantitative analysis of the ligaments from groups I to IV using the light microscope.

	Group I: Control/Right MCL n = 10	Group I: Control/Left MCL n = 10	Group II: Control+M Right MCL n = 10	Group II: Control+M Left MCL n = 10	Group III: DM/Right MCL n = 10	Group III: DM/Left MCL n = 10	Group IV: DM+M/Right MCL n = 10	Group IV: DM+M/Left MCL n = 10
Characteristic pattern of the tissue	10	10	10^a^	8^a^	4^a^	5^a^	9^b^	8^b^
Lymphocytic infiltration	1	1	0^a^	1^a^	2^a^	5^a^	2^b^	3^b^
Loosened structure	0	2	1^a^	1^a^	4^a^	8^a^	4^b^	2^b^
Mast cell infiltration	0	0	0^a^	2^a^	4^a^	3^a^	3^b^	1^b^
Increased fibroblasts	0	0	1^a^	2^a^	4^a^	5^a^	5^b^	2^b^
Bad quality assessed optically: hypertrophy, fibroblast hyperactivity, and hypercellularity	0	2	1^a^	0^a^	5^a^	10^a^	1^b^	2^b^

Abbreviations: Control, control group; Control + M, control group + melatonin supplementation; DM, diabetic group; DM + M, diabetic group + melatonin supplementation; Right MCL, sham procedure was performed; Left MCL, incision of the ligament was performed. Data are expressed as mean ± SD. ^a^ *p* < 0.05 versus the control group; ^b^ *p* < 0.05 versus the diabetic group.

Histopathological screening of the left MCL from group III with DM revealed a loosened structure of the connective tissue (8; *p* < 0.05) together with lymphocytic infiltration (5; *p* < 0.05), increased quantities of fibroblasts (5; *p* < 0.05) and mast cell infiltration (3; *p* < 0.05) and features of increased fibroblast activity influencing the overall optically assessed quality of the tissue. Overall hypertrophy of the tissue, hypercellularity, and invalid fibroblast function were described in half of the samples (5; *p* < 0.05) compared to group I.

A minority of the right MCL samples from group III presented the characteristic pattern of the tissue (4; *p* < 0.05), with increased fibroblasts (4; *p* < 0.05), mast cell infiltration (4; *p* < 0.05), loosened structure (4; *p* < 0.05), and lymphocytic infiltration (2; *p* < 0.05). Overall hypertrophy of the tissue, hypercellularity, and invalid fibroblast function were described in half of the samples (5; *p* < 0.05) compared to group I.

Nine samples from group IV, animals with DM and Mel supplementation, presented with a characteristic pattern of the right MCL, which is statistically significant compared to group II of animals without DM and with Mel supplementation (*p* <0.05). Lymphocytic infiltration appeared in two samples (*p* < 0.05), and increased fibroblasts (5; *p* < 0.05), mast cell infiltration (3; *p* < 0.05), and loosened structure (4; *p* < 0.05) appeared in other samples. Only one sample was assessed as having bad quality (*p* < 0.05) compared to the corresponding group II. 

Eight of the left MCL samples from group IV presented with the characteristic pattern of the right MCL, which is statistically significant compared to group II (*p* < 0.05). Lymphocytic infiltration appeared in three samples (*p* < 0.05), and increased fibroblasts (2; *p* < 0.05), mast cell infiltration (1; *p* < 0.05), and loosened structure (2; *p* < 0.05) appeared in others. The quality of two samples was assessed as bad compared to group II (*p* < 0.05).

All the left MCL samples from group III, animals with DM, seemed to be disintegrated and frayed. Their elasticity was consistent (90%; *p* = 0.001). Some samples were found to be mildly hypertrophic (2 ± 0.92; *p* = 0.001), and some were intensively hypertrophic (6 ± 0.22; *p* = 0.001). The right MCL samples from group III seemed hypercellular (8; *p* > 0.05) and showed lymphocytic infiltration (2; *p* = 0.00865). However, the structure differed from the physiological appearance of the ligament tissue. Numerous collagen fibers seemed loosened with mast cell infiltrations in some cases (4; *p* = 0.8567). The overall screening revealed significantly better qualitative measures in group IV, the melatonin-supplemented diabetic group, compared to the values from group III, with diabetes (*p* = 0.01).

The process of dissection facilitates morphological and histological changes in the appearance of the MCL. The glistening white appearance under the microscope was not no longer recognizable. DM was found to be a cofactor for exacerbating the change. Severely affected fragments were grayish and amorphous, disproportionally distinguished into fusiform or nodular thickening portions. Compared to group III, the melatonin supplementation (group IV) brought statistically significant improvement in all the morphological and histological measures, *p* < 0.05. Melatonin supplementation in group II of animals without DM and with Mel supplementation diminished elastin fiber loss compared to the control group from 0.5 ± 0.2, *p* < 0.05 and 0, *p* < 0.05 for the right MCL samples from group I and the right MCL samples from group II, respectively. The same observation was made about the left MCL samples from group I, which had a score of 1.05 ± 0.55, *p* < 0.05, and the left MCL samples from group II, which had a score of 0. Melatonin supplementation in group II of animals without DM and with Mel supplementation did not cause any significant changes in other parameters.

### 3.3 Laboratory tests: blood plasma assessment of molecule concentration

Blood samples were drawn from the experimental animals in all groups (I–IV). In diabetic rats from group III, blood glucose, total lipids, cholesterol, and triglyceride presented significantly increased values (*p* < 0.05) compared to control animals (Table 4). However, group IV, the diabetic group that received melatonin, presented with reduced concentrations of blood glucose, triglyceride, total lipids, and cholesterol (*p* < 0.05).

**TABLE 4:** Fasting blood glucose levels before (Leong et al., 2020) and after (Longo et al., 2009) STZ injection, total lipids (Nelson et al., 2006), triglyceride levels (Fleming et al., 2005), and cholesterol levels (Fleming, 2003) represented through statistical analysis of the different experimental groups.

		Group I: Control n = 10	Group II: Control+M n = 10	Group III: DM n = 10	Group IV: DM+M n = 10	*p*-value
1	Fasting blood glucose before STZ injection (mg/dL)	93 (±7)	91 (±3)^a^	90 (±13)^a^	95 (±6)^b^	0.0022
2	Fasting blood glucose after STZ (or vehicle) injection (mg/dL)	99 (±5)	102 (±11)	258 (±38)^a^	154 (±19)^b^	0.0017
3	Total lipid (mg/dL)	470 (±25)	420 (±55)^a^	650 (±50)^a^	375 (±35)^b^	<0.001
4	Triglyceride levels (mmol/L)	1.5 (±0.12)	1.65 (±0.35)^a^	2.75 (±0.25)^a^	1.5 (±0.2)^b^	0.0047
5	Cholesterol levels (mmol/L)	2.65 (±0.15)	3.22 (±0.18)^a^	6.2 (±0.6)^a^	3.18 (0.2)^b^	<0.001

Abbreviations: Control, control group; Control + M, control group + melatonin supplementation; DM, diabetic group; DM + M, diabetic group + melatonin supplementation. Data are expressed as mean ± SD. ^a^ *p* < 0.05 versus the control group; ^b^ *p* < 0.05 versus the diabetic group.

Group II, which received melatonin, did not show any significant changes in any of the measured parameters compared to the healthy control group I. Fasting blood glucose levels from group III, the untreated DM group, increased significantly compared to the healthy control. Group IV, the DM+M group, showed higher fasting blood glucose levels than group I but significantly lower than those in group III, the DM group, at 258 ± 38 and 154 ± 19; *p* < 0.05, respectively.

All total lipid, triglyceride levels, and cholesterol levels increased significantly in non-treated diabetic group III, at 650 ± 50, 2.75 ± 0.25, and 6.2 ± 0.6, respectively, compared to healthy controls, at 470 ± 25, 1.5 ± 0.12, and 2.65 ± 0.15 for total lipids, triglycerides, and cholesterol, respectively, as shown in Table 5. A significant decline in total lipids, triglyceride levels, and cholesterol levels was observed in the melatonin-treated diabetic group IV (375 ± 35, 1.5 ± 0.2, 3.18 ± 0.2, respectively) compared to the non-treated diabetic group.

**TABLE 5:** Levels of lipid peroxides, nitric oxide, and antioxidants in plasma of rats.

	Control n = 10	Control+M n = 10	DM n = 10	DM+M n = 10	*p*-value
LPO (nmol/mL)	0.28 (±0.01)	0.33 (±0.1)^a^	0.65 (±0.15)^a^	0.16 (±0.01)^b^	0.0011
Nitric oxide (ng/mL)	137.28 (±12.25)	132.55 (±7.5)^b^	271.63 (±16.5)^a^	142.65 (±30.45)^b^	0.0567
Total thiols (nmol/mL)	0.98 (±0.11)	1.13 (±0.46)^a^	0.32 (±0.08)^a^	1.24 (±0.19)^b^	0.9827
SOD (ng/mL)	128.72 (±15.45)	114.85 (±14.13)^a^	102.36 (±10.05)^a^	140.65 (±8.26)^b^	<0.001
Ceruloplasmin (mg/L)	161.65 (±10.58)	176.37 (±11.5)^a^	102.84 (±10.91)^a^	216.22 (±9.96)^b^	0.1765
Albumin (g/L)	43.18 (±2.17)	39.96 (±3.15)^a^	29.37 (±4.56)^a^	39.28 (±1.99)^b^	<0.001
Uric acid (mmol/L)	0.56 (±0.45)	0.49 (±0.23)^a^	0.71 (±0.4)^a^	0.51 (±0.03)^b^	0.0321

Abbreviations: Control, control group; Control + M, control group + melatonin supplementation; DM, diabetic group; DM + M, diabetic group + melatonin supplementation. Data are expressed as mean ± SD. ^a^ *p* < 0.05 versus the control group; ^b^ *p* < 0.05 versus the diabetic group.

### 3.4 Laboratory tests: oxidative stress markers in blood plasma

Lipid peroxides and uric acid showed increased concentration in group III (*p* < 0.05), and reduced albumin concentration was found in group III (*p* < 0.05) (Table 5). Nitric oxide was also increased (*p* > 0.05) in group III, while total thiol and ceruloplasmin were reduced (*p* > 0.05) when compared to the group I. Group II significantly increased total thiol and ceruloplasmin activity. In addition, group II significantly decreased (*p* < 0.001, *p* < 0.05) lipid peroxides and uric acid, respectively. Rats group II only showed a decrease (*p* > 0.05) in nitric oxide. Group IV showed decreased values of LPO, NO and uric acid compared to group III. However the melatonin supplementation in group IV increased the results of total thiols, SOD, ceruroplasmin and albumin concentration in comarison to group III.

### 3.5 Laboratory tests: oxidative status of ligamentous homogenates

Ligament harvesting included samples for homogenization. The probes of ligament homogenates showed a significant increase in lipid peroxides in diabetic rats compared to the controls (*p* < 0.05) (Table 6). GST activities increased significantly (*p* < 0.001) in group IV, diabetic rats treated with melatonin. Total thiols presented with similar values (*p* < 0.05) in group II, rats treated with melatonin, and group I, the control group. Samples from group IV rats treated with melatonin showed significantly increased (*p* < 0.05, *p* < 0.001) activities of superoxide dismutase and catalase, respectively, compared to the samples from group III rats with DM and showed a similar range of values compared to the group I control.

**TABLE 6:** Levels of lipid peroxides (LPO), total thiols, and the activities of superoxide dismutase (SOD), catalase, and glutathione S-transferase (GST) in the ligament homogenates of the different experimental groups.

		Control n = 10	Control+M n = 10	DM n = 10	DM+M n = 10	*p*-value
1	LPO (pmol/mg Hb)	11.28 (±2.27)	12.01 (±1.5)^a^	22.65 (±4.65)^a^	10.16 (±1.12)^b^	0.0032
2	Total thiols (nmol/mg Hb)	22.68 (±1.81)	24.16 (±2.47)^a^	14.82 (±2.38)^a^	25.64 (±3.19)^b^	0.0727
3	GST (nmol/min/mg Hb)	224.7 (±35.5)	222.5 (±27.9)^a^	162.28 (±20.46)^a^	298.25 (±35.56)^b^	<0.001
4	SOD (ng/mg Hb)	5.65 (±0.5)	4.25 (±0.73)^a^	2.99 (±0.83)^a^	5.58 (±0.84)^b^	0.0032
5	Catalase (U/mg Hb)	0.65 (±0.12)	0.72 (±0.18)^a^	0.29 (±0.16)^a^	0.61 (±0.09)^b^	<0.001

Abbreviations: Control, control group; Control + M, control group + melatonin supplementation; DM, diabetic group; DM + M, diabetic group + melatonin supplementation. Data are expressed as mean ± SD. ^a^ *p* < 0.05 versus the control group; ^b^ *p* < 0.05 versus the diabetic group.

**FIGURE 5:**
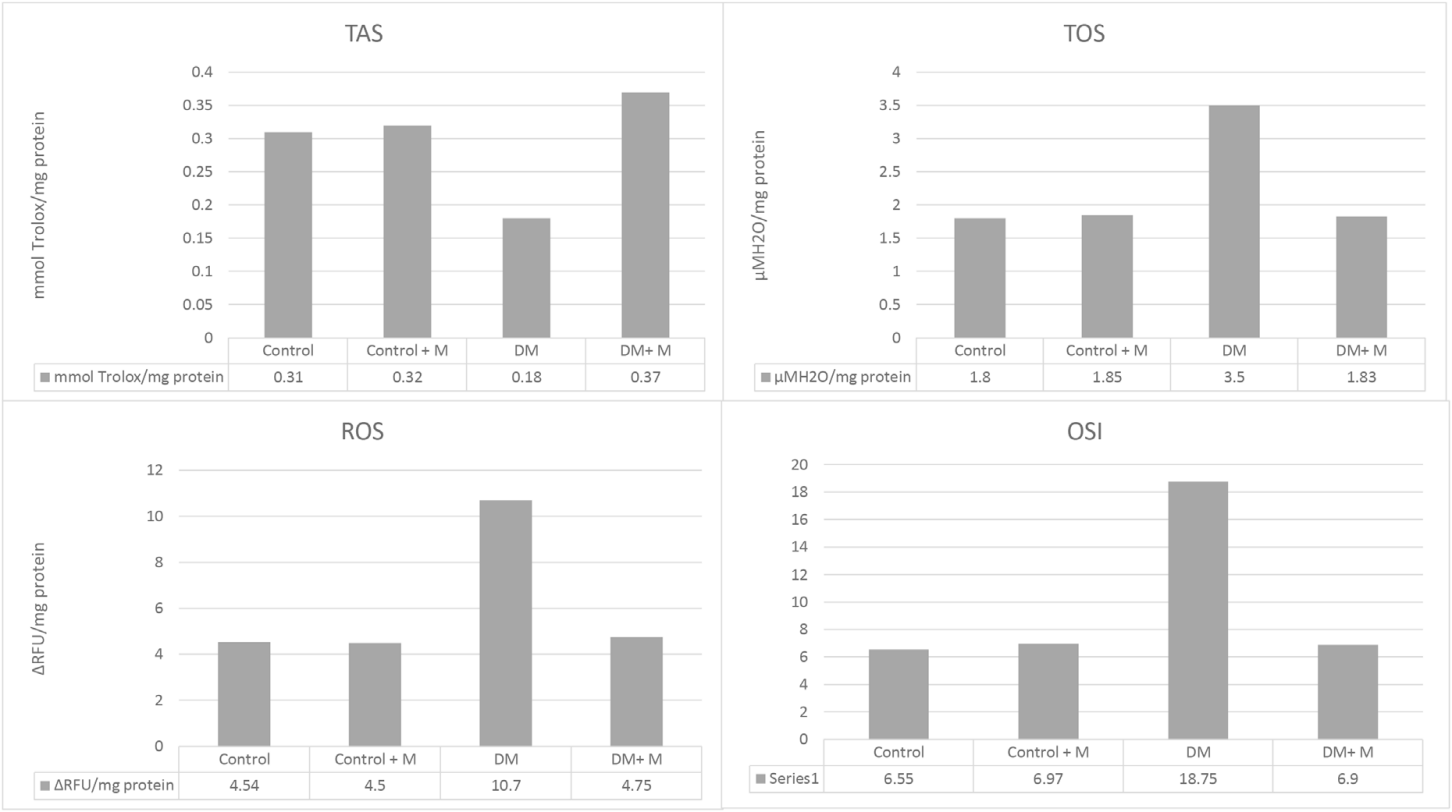
Ligament homogenate TAS, TOS, ROS levels, and OSI rates of all groups. Abbreviations: Control, control group; Control + M, control group + melatonin supplementation; DM, diabetic group; DM + M, diabetic group + melatonin supplementation; OSI, oxidative stress index; ROS, reactive oxygen species; TAS, total antioxidant status; TOS, total oxidant status. Abundance of groups: Control: n = 10; Control + M: n = 10; DM: n = 10; DM + M: n = 10. Data are expressed as mean ± SD. ^a^ *p* < 0.05 versus the control group; ^b^ *p* < 0.05 versus the diabetic group.

Oxidative and antioxidative status were measured among all the groups (I–IV) (Figure 5). In the diabetic groups, it was found that TAS levels decreased while TOS, ROS levels, and OSI rates increased. These changes were found to be meaningful when compared to the control group (*p* < 0.0001, *p* < 0.0001, *p* < 0.0001, and *p* < 0.0001, respectively). Treatment with melatonin in diabetic rats mitigated these changes (*p* < 0.0001, *p* < 0.0001, *p* < 0.0001, *p* < 0.0001, respectively).

**FIGURE 6:**
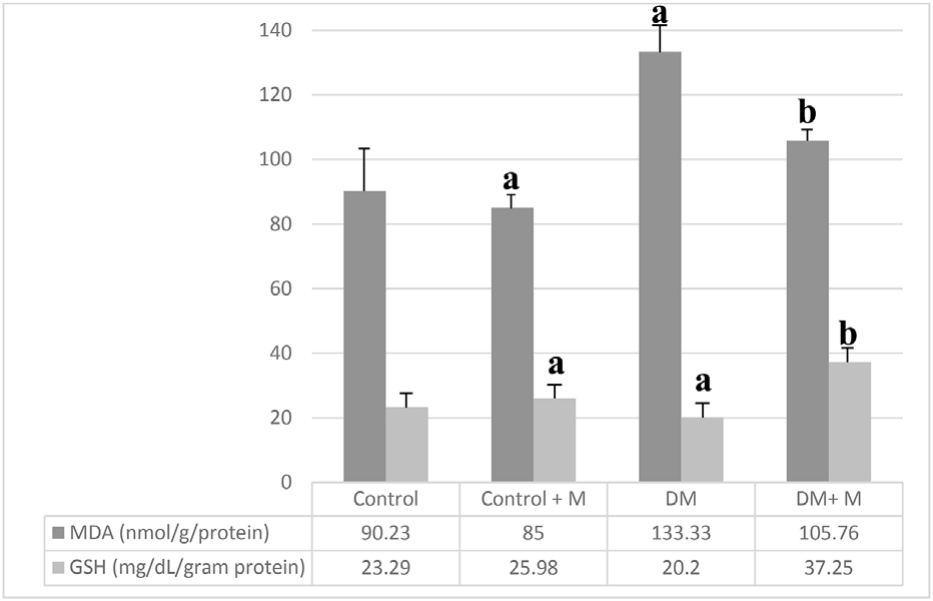
Levels of MDA and GSH in the various ligament homogenates. Abbreviations: Control, control group; Control + M, control group + melatonin supplementation; DM, diabetic group; DM + M, diabetic group + melatonin supplementation; MDA, malondialdehyde; GSH, glutathione. Data are expressed as mean ± SD. ^a^ *p* < 0.05 versus the control group; ^b^ *p* < 0.05 versus the diabetic group.

MDA and GSH concentrations were measured (Figure 6). The highest MDA value in the ligament homogenates was obtained in group III, the DM group. MDA levels in the bone tissue in group IV, with DM and melatonin supplementation, were lower than in group III, the DM group, but higher than in all other groups. The GSH values appeared higher in the melatonin-supplemented groups than in groups without supplementation. The highest GSH levels were measured in group IV, the diabetic group supplemented with melatonin.

## 6 Discussion

Many factors are responsible for the successful healing and structural recovery of injured ligaments. Uncorrectable risk factors for poor healing potential after trauma or surgery are comorbidities, muscle degeneration and atrophy, autoimmunological diseases, larger tear size, poor soft tissue quality, repetitive trauma, smoking, and diabetes (Loots et al., 1999).

The results of our study evidence the high grade of morphological changes occurring in the ligaments in a hyperglycemic state. We confirm a positive relationship between hyperglycemia and immune response, inflammation, and extracellular matrix synthesis. In addition, high glucose levels have been shown to inhibit the proliferation and regeneration of ligament fibroblast cells, which might partly account for delayed regeneration and healing in animals with diabetes (Furnary et al., 1999; Damasceno et al., 2014a).

Ligaments play a significant role in stabilizing the body and carrying mechanical forces (Yamaguchi et al., 2006; Shemesh et al., 2018; Adamska et al., 2022).

The enhanced concentration of collagen cross-linking stimulated in inflammatory conditions of DM worsens the quality of tissue and disables its significant roles in the functioning of the body (Al-Mulla et al., 2011; Kato et al., 2015; Wilkinson et al., 2019a; Hippensteel et al., 2019).

Our study posits that DM significantly enables the physiological response to either stress or trauma. We provide evidence of the mechanisms responsible for the alteration of antioxidant defense. Furthermore, identifying specific molecular markers for ligament progenitor cells in adult tissues might help elucidate their contribution to ligament homeostasis and regeneration and would allow treatment of some of the complications of diabetes. Asahara et al. (2017) hypothesized that Egr-1 expression may be compromised in DM, leading to impaired collateral vessel growth and impairing healing and homeostasis. This evidence shows that DM provokes pathological tissue responses through multiple mechanisms.

Findings show a decreased proliferation rate, higher turnover, and breakdown of fibroblasts derived from soft tissues induced by DM (Lerman et al., 2003). The results from the histopathological examination of samples from rats with DM in groups III and IV presented a disintegrated structure, diminished fibroblast functioning, and hypercellularity, enabling physiological response to trauma. These suggest that excessive proliferation and an altered structure of the fibroblasts may contribute to poor production of the main component of ligaments, namely, collagen, and thus account for their impaired healing (Lerman et al., 2003; Ko et al., 2021).

Studies found that DM delays oral wound healing, which agrees with our finding of peripheral ligaments (Ko et al., 2021). Diabetic wounds were exacerbated by increased inflammation, as noted by greater numbers of neutrophils, enhanced re-epithelialization, and increased levels of TNF compared to wounds in nondiabetic patients (Vicente et al., 2021). In skin wounds, DM contributes to prolonged inflammation (Wang and Graves, 2020).

Ligament wounds have reduced angiogenic response compared to skin wounds in general because of excessive inflammation (Wilkinson et al., 2019b). Jeon et al. (2021) promote the primary benefit of inflammatory pathway inhibition in early phases of wound healing by enhanced production of connective tissue matrix, increased myofibroblast formation, and greater angiogenesis in diabetic wounds. This is important because reducing prolonged inflammation improves the healing of wounds in type 2 DM animals. Thulasingam et al. (2019) confirm that normoglycemia prevents complications and comorbidities in healthy mice and can reverse degeneration in DM mice. Our study confirmed that animals from normoglycemic groups I and II presented with better overall antioxidative status, and, in contrast, groups III and IV, namely, animals with DM, had high oxidative status values.

Endoplasmic reticulum (ER) stress mainly involves transcription factors. Although moderate ER stress can effectively protect the body, excessive ER stress causes degeneration, as happens during DM involvement (Ruan et al., 2021). Song et al. (2021) evidenced ER effects in apoptosis of the periodontal ligament (PDL) and vascular calcification in a rat model. Furthermore, our study confirms that DM also provokes those complications in the MCL.

There is evidence that streptozotocin-induced diabetes releases free radicals (Damasceno et al., 2014b). As glutathione is closely linked to glucose metabolism via NADPH, it is logical that free radical metabolism is altered in diabetes (Johansen et al., 2005).

In the present experiment, streptozotocin treatment caused a significant increase in lipid peroxidation in plasma. Increased glycation of collagen and plasma proteins in diabetes may stimulate the oxidation of lipids, which in turn may stimulate autooxidative reactions of sugars, enhancing damage to both lipids and proteins in the circulation and continuing the cycle of oxidative stress (Volpe et al., 2018; Ito et al., 2019).

It is evident from the present study that melatonin supplementation may help in the prevention and protection against the production of free radicals in diabetes. The antioxidant effects of melatonin caused a marked elevation in the antioxidant system activity and reduced oxidative stress.

Nitric oxide is produced in the endocrine pancreas and contributes to the synthesis and secretion of insulin. The potential role of NO in insulin secretion is disputable—both stimulatory and inhibitory effects have been reported (Gheibi and Ghasemi, 2020). In the present study, plasma nitrite as an end product of nitric oxide activity was elevated in the untreated diabetic rats. Similar results have been previously obtained in other studies (Anggård, 1994; Welsh et al., 1994; Nagareddy et al., 2006; Pacher et al., 2007).

Uric acid is considered a non-enzymatic antioxidant, but increased production of uric acid means increased free radical production due to activation of the xanthine oxidase enzyme system (Sekizuka, 2022). In our experiment, uric acid levels were increased in diabetic rats. In the present study, streptozotocin treatment caused a significant depletion of both enzymatic and non-enzymatic antioxidants in plasma or erythrocyte lysates or the homogenates of both ligaments and muscles. Accordingly, SOD treatment can protect *in vivo* or *in vitro* against the high toxic potential of the superoxide radicals in alloxan-induced diabetic rats (Gandy et al., 1982; Najafi et al., 2021). A significant decrease in plasma ceruloplasmin and albumin was also found. However, uric acid increased. In our experiment, melatonin treatment generally normalized oxidative stress in streptozotocin-induced diabetic rats. Melatonin treatment reduces oxidative stress (lipid peroxidation, nitric oxide, and uric acid) while elevating enzymatic and non-enzymatic antioxidant systems in blood, ligaments, and muscle homogenates. Anwar et al. reported that melatonin had a potent reducing effect on the production of lipid peroxides in rats exposed to cytotoxic drugs (Anwar and Meki, 2003). Nitric oxide, involved in the neuropathy that is one of the complications of diabetes as a result of oxidative stress, is reduced after melatonin treatment (Sáenz et al., 2002; Huang et al., 2015; Bicer et al., 2018; Hosseini et al., 2022). Storr et al. (2002) reported that melatonin inhibited nitric oxide synthase enzyme and reduced nitric oxide production. The reduction in uric acid levels after melatonin treatment may be due to the reduction of lipid peroxidation, triglycerides, and cholesterol, while the elevation of these substances may increase uric acid synthesis (Ferlazzo et al., 2020).

According to the following articles, the highest MDA levels in ligament tissues were found in diabetic groups that were not supplemented with melatonin. This result is consistent with literature, which states that oxidant stress that increased in diabetes had a negative impact on bone metabolism. What is more, it was reported that endurance exercise was able to enhance bone quality by restoring bone blood flow (Atalay et al., 1997; Hara et al., 1997; Turgut et al., 2003; Bae et al., 2018; Zhao et al., 2022; Marcucci et al., 2023).

However, those results differ from the results of a study conducted by Bicer et al. (2018). This study interestingly found continuously high bone MDA levels in the diabetic group despite subjecting them to swimming exercises. Those elevated MDA values that were established in the bone tissue demonstrate that the lipid peroxidation that is enhanced in diabetic rats cannot be offset by exercise.

The abovementioned results suggest that MDA values in the bone tissue in group IV, diabetic rats supplemented with melatonin, should be lower than the values in the diabetic group without supplementation but higher than those in all other groups. The results of the current study indicate that melatonin supplementation suppresses MDA values in diabetic groups. It was reported that melatonin had a protective effect on tissues throughout the body (Bazyar et al., 2021) and prevented lipid peroxidation in the bone tissue (Bazyar et al., 2022; Dludla et al., 2023). The current results confirm that it presents the same protective effects in bones. Group IV, the melatonin-supplemented diabetic group, had the highest bone tissue GSH values. This result demonstrates that melatonin supplementation significantly increases GSH levels in diabetic rats. Melatonin supplementation was also reported to significantly increase antioxidant activity in rats with induced diabetes.

The most pronounced effect of melatonin administration was the prevention of an increase in NO levels in blood plasma during streptozotocin-induced diabetes, which implies that melatonin may operate as an NO scavenger and carrier.

The biochemical analyses done in the present study prove the existence of oxidative stress in diabetes. These parameters are ROS, TOS, TAS, and OSI, and they indicate the level of accumulated oxidants and possible tissue damage caused by DM. The typical pathophysiology of oxidative stress provokes an ROS and TOS increase and a TAS level decrease. It is explained by TAS activity in the effective reduction of oxidative stress complications, and, furthermore, it shows an increased antioxidant level. The results are in accordance with the literature findings (Rehman and Akash, 2017; Hu et al., 2022).

In addition, melatonin diminishes the levels of oxidative stress in organisms directly by inducing the antioxidant defense systems (Görgün et al., 2002; Ghanbari et al., 2016; Raygan et al., 2019; Ertik et al., 2023). Melatonin can decrease oxidative stress by stimulating the gene expression of antioxidant enzymes such as SOD and GSH-Px (Abdulwahab et al., 2021; Grabia et al., 2023; Mirapoğlu et al., 2023; Rahbarghazi et al., 2023). Ahmad et al. (2018) found that 1 mg/kg of melatonin given to the diabetic group IV increased the GSH level, CAT, and SOD activities but decreased the LPO level in the pancreas. Albazal et al. (2021) found that the GSH levels and CAT and SOD activities decreased in diabetic animals, while LPO and ROS levels increased. Treatment with melatonin at 10 mg/kg/day for 7 weeks reversed the negative impact of oxidative stress (Ahmad et al., 2018). Another study reported that administration of 10 mg/kg melatonin to diabetic rats for 14 days improved oxidative stress parameters significantly (Albazal et al., 2021; Kahya and Nazıroğlu, 2016). In the diabetic group, GSH, TAS levels, and GPx activity diminished, while LPO, ROS, and TOS levels increased. It was observed that oxidative stress parameters were reversed upon administering melatonin to diabetic rats by reducing oxidative stress (Kahya and Nazıroğlu, 2016). The present study examined oxidative stress parameters such as TAS, TOS, ROS, and OSI. It was found that the oxidative stress in the bone tissue caused by diabetes decreased upon the administration of melatonin for 4 weeks. When both the effect of melatonin on antioxidant enzymes and the free radical scavenging effect are considered, the outcomes are compatible with previous reports.

Melatonin has a pleiotropic effect on the organism. Because many organs have melatonin receptors, it is a potent antioxidant, anti-inflammatory, free radical scavenger, and helpful in fighting inflammatory diseases (Guan et al., 2022). Melatonin receptors might have therapeutic potential as they belong to the G protein-coupled receptor superfamily and, therefore, are abundant throughout the organism. Understanding the role of melatonin and its receptors on glucose homeostasis is urgent because of the wide use of melatonin for many indications, either as a prescribed medication or as a supplement without medical prescription, in many countries (Gomes et al., 2021). The antagonistic relationship between melatonin and insulin defines its influence in regulating insulin secretion and action. Melatonin treatment improved glucose homeostasis, energy balance, and overall health in diabetes mellitus. Literature reports melatonin’s cytoprotective role as an antioxidant and free radical scavenger in combating oxidative stress, preserving beta-cell function, and influencing the development of diabetic complications (Wajid et al., 2020).

In the present study, an increased level of circulating melatonin was associated with a decrease in blood glucose level, reducing the pro-oxidant susceptibility and pro-inflammatory responses. The role played by melatonin on the endocrine system, including insulin metabolism and proving the presence of melatonin receptors in many different organs, allows us to assume that the processes taking place during regeneration, for example, at night when melatonin secretion is the highest, are not a coincidence, and that melatonin is a hormone that affects reactions regulating homeostasis (Guan et al., 2022). However, with age, the concentration of melatonin decreases, and its role becomes less important. Therefore, many researchers use exogenous melatonin to demonstrate a protective effect on circadian rhythm, immune system, response to inflammation, wound healing, and tissue regeneration (Karamitri and Jockers, 2019).

We aimed to evaluate the effect of melatonin administration in STZ-induced diabetic rats and healthy rats. Melatonin administration in STZ-induced diabetic rats reduced hyperglycemia, hyperlipidemia, and oxidative stress. Melatonin acted as an anti-inflammatory agent that reduced pro-inflammatory mechanisms and oxidative stress biomarkers, such as MDA, GSH, and NO. Melatonin succeeded in enhancing ligament cell regeneration under severe inflammation caused by surgical conditions and injury.

The authors of a study evaluating the role of melatonin in foot ulcer regeneration underlined its potential in reducing the risk of hyperglycemia, neuropathy, vascular damage, and immune system impairment in diabetic patients. All those characteristics are proven to play a role in ligament healing. Correspondingly, melatonin use brings promising results in diabetic patients with joint and soft tissue complications (Sajjadpour et al., 2023).

The results of the present study indicated that melatonin supplementation prevents increased free radical production and inhibits antioxidant activity resulting from diabetes in the bone tissue. Melatonin has many positive effects and antioxidant properties. In the present study, the effect of melatonin on diabetes was examined using different biochemical parameters. It was observed that melatonin had a healing effect and restored normal biochemical parameters. The data indicate that the use of melatonin with or without insulin may be effective in preventing or at least retarding the development of some diabetic complications.

### 6.1 Summary

Surgical intervention within the ligament contributes to the impaired healing potential and loss of the morphological structure in the group of animals with induced diabetes.

Inflammatory changes caused by autooxidative activities are high in diabetic groups. Melatonin supplementation in a group of animals with induced diabetes contributed to an increase in the concentration of antioxidants, providing a protective effect on the ligaments in conditions of hyperglycemia.

These findings evidence the degenerative influence of DM on the ligament tissue, inhibiting physiological protective mechanisms for oxidative stress. Treatment with melatonin in diabetic rats mitigated alterations and improved the antioxidant status of ligaments in the diabetic group.

## Data Availability

The raw data supporting the conclusions of this article will be made available by the authors, without undue reservation.
